# Effects of different antioxidants and their combinations on the oxidative stability of DHA algae oil and walnut oil

**DOI:** 10.1002/fsn3.2883

**Published:** 2022-04-11

**Authors:** Xue‐Chen Pei, Fa‐Wen Yin, Xu Zhong, Hui‐Lin Liu, Liang Song, Guan‐Hua Zhao, Yong‐Fu Wang, Da‐Yong Zhou

**Affiliations:** ^1^ 12400 School of Food Science and Technology Dalian Polytechnic University Dalian China; ^2^ National Engineering Research Center of Seafood Dalian China; ^3^ Collaborative Innovation Center of Seafood Deep Processing Dalian China; ^4^ QingdaoSeawit Life Science Co., Ltd Qingdao China

**Keywords:** accelerated storage, DHA algae oil, shelf life prediction, tea polyphenol palmitate, walnut oil

## Abstract

Through monitoring Rancimat induction time (RIT), peroxide value (POV), and thiobarbituric acid‐reactive substances (TBARS) of docosahexaenoic acid (DHA) algae oil and walnut oil during accelerated storage, the effects of the single and the combinations of seven kinds of antioxidants involving ascorbyl palmitate (AP), phytic acid (PA), vitamin E (VE), antioxidant of bamboo leaves (AOB), rosemary extract, tea polyphenols (TP), and tea polyphenol palmitate (TPP) against lipid oxidation were evaluated. RIT, POV, and TBARS results showed that the DHA algae oil sample containing 600 mg/kg TPP revealed the strongest stability and the walnut oil sample containing 450 mg/kg TPP and 100 mg/kg TP revealed the strongest stability. Then, the shelf lives of two oils were predicted from the extrapolation of the linear regression model between Log RIT and temperature. Our results indicated that the optimal antioxidant could prolong the shelf lives of DHA algae oil and walnut oil by 2.31‐ and 7.74‐fold, respectively.

## Graphical abstract

Docosahexaenoic acid (DHA) algae oil containing tea polyphenol palmitate (TPP) was the most stable to oxidation. Walnut oil containing TPP + TP (tea polyphenols) was the most stable to oxidation. The single TPP extended 2.31‐fold shelf life of DHA algae oil and the combination of TPP + TP extended 7.74‐fold shelf life of walnut oil.

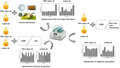

## 1 INTRODUCTION

As one of the three essential nutrients for human life, lipid is mainly used in cooking to improve the color and taste of food in the cooking process (Zhou et al., 2020). It can also be used as healthcare product to supply essential fatty acids such as polyunsaturated fatty acids (PUFAs). PUFAs, especially omega‐3 (n‐3) PUFAs, have numerous functions in human health including preventing cardiovascular and inflammatory disorders, lowering the incidence of mental diseases, and enhancing the brain's memory (Castejon & Señoráns, 2020). However, oxidation of PUFAs is a major problem in the storage of lipids and lipid‐based products, which will impart unacceptable aromas, damage the nutritional quality of food, and cause the production of toxic compounds (Arab‐Tehrany et al., 2012).

For retarding lipid oxidation, besides limiting oxidation‐promoting factors such as light, oxygen, and high temperatures, the addition of antioxidants and the microencapsulation technology are commonly adapted as well (Ghnimi et al., 2017; Großhagauer et al., 2019). Especially, adding various antioxidants or antioxidant combinations has become an inexpensive and high‐efficient method (Gulcin, 2020). Many researchers have concentrated on screening the optimal antioxidant for stabilizing different kinds of lipids. For instance, Yin et al. (2021) revealed that the natural antioxidant mixture composed of 0.0224% (w/w) vitamin E (VE), 0.0259% rosemary extract (RE), and 0.0166% ascorbyl palmitate (AP) showed the best antioxidant ability for protecting the commercial microalgal DHA‐rich oil. Lu et al. (2020) proposed that the composite antioxidants composed of 240 mg/kg tea polyphenol palmitate (TPP), 40 mg/kg phytic acid (PA), 80 mg/kg tocopherol, and 40 mg/kg AP showed the strongest effectiveness in retarding flaxseed oil's oxidation.

For the purpose of estimating the inhibitory effects of antioxidants against lipid oxidation, several chemical methods like peroxide value (POV), thiobarbituric acid‐reactive substances (TBARS), or *p*‐anisidine value as well as physical methods like conjugated diene assay, fluorescence, or infrared spectroscopy are available for determining the degree of lipid oxidation (Abeyrathne et al., 2021; Miguel, 2010). Considering the importance of saving time industrially, Rancimat test is becoming popular for the determination of oxidative stability of the oil products, which only considers three operational factors including sample quantity, temperature, and flow rate of air (Kurtulbaş et al., 2018). In particular, compared with traditional chemical or physical methods, it is easier to operate and is more environmentally friendly without using organic solvents (Félix‐Palomares & Donis‐González, 2021). In recent years, researchers have used this method to predict the shelf life of oils. For example, Ghosh et al. (2018) predicted the shelf lives of the blends of sunflower and sesame oil with different ratios using the Rancimat test. Aktar and Adal (2019) used Rancimat test to predict the shelf life of avocado oil at 25℃.

Docosahexaenoic acid (DHA, C22:6 n‐3) algae oil, a major resource of long‐chain n‐3 PUFAs, particularly DHA (C22:6 n‐3), becomes a new focus due to its health benefits (Castejon & Señoráns, 2020; Chen et al., 2016). And walnut oil, mainly comprised of linoleic acid (C18:2 n‐6), is unique due to its ideal balance of n‐6 PUFAs and n‐3 PUFAs (a 4:1 ratio; Nguyen & Vu, 2021; Zhou et al., 2016). Up to now, few studies have focused on screening the suitable antioxidant combinations among individual antioxidants and their mixtures for both DHA algae oil and walnut oil. Therefore, in this research, Rancimat test was used to obtain the optimal antioxidant combinations from AP, PA, VE, antioxidant of bamboo leaves (AOB), RE, tea polyphenols (TP), and TPP for stabilizing DHA algae oil and walnut oil, as well as to predict the shelf lives of the oils containing the optimal antioxidant combinations for the first time. This report provides an easy and efficient design process for screening suitable composite antioxidants for edible oils using Rancimat and can serve as a reference for similar research.

## 2 MATERIALS AND METHODS

### 2.1 Materials

Refined DHA algae oil and walnut oil (with no antioxidants) were purchased from Qingdao Seawit Life Science Co., Ltd. Seven natural antioxidants involving AP, PA, VE, AOB, RE, TP, and TPP were also obtained from Qingdao Seawit Life Science Co., Ltd.

### 2.2 Sample preparation

Types and amounts of additive single antioxidants and their binary mixtures can be seen in Table S1. Based on the Chinese Standard GB, 2760‐2014, the additive amount of each single antioxidant including AP, PA, VE, AOB, RE, TP, and TPP was the maximum allowable amount. For the binary mixtures comprised of TPP and one of the other six antioxidants, the additive amount of each antioxidant was half of the maximum allowable amount. And in order to optimize the blend ratio of the optimal binary antioxidant, the additive amounts of TPP were 1/3, 2/3, 1/4, 3/4, 1/5, 4/5, 2/5, or 3/5 of its maximum allowable amount, while the corresponding additive amounts of TP were 2/3, 1/3, 3/4, 1/4, 4/5, or 1/5 of its maximum allowable additive amount, respectively.

For the determination of POV and TBARS, all the oil samples were prepared in dark containers and saved in an air‐circulating oven (the operating temperature: 60℃). Briefly, the samples were taken at fixed time intervals of 1 day until 3 days.

### 2.3 Measurement of Rancimat induction time

The RIT values of oil samples were acquired using a Rancimat 892 apparatus (Metrohm) based on Liu et al. (2018). The Rancimat test was executed with 3.0 g of oil sample at a temperature of 110℃ and at an air flow rate of 20 L/h.

### 2.4 Measurement of the peroxide value

The POVs of oil samples were determined based on the Chinese Standard GB/T 5009.37‐2003 and a previous study (Folch et al., 1957). In brief, 0.1 g of oil sample was reacted with 10 μl ferrous ion and 10 μl ammonium ion in the dark for 20 min, then, the supernatant's absorbance was read at 510 nm. The POVs of oil samples were calculated by the standard curve of hydrogen peroxide isopropylbenzene.

### 2.5 Measurement of thiobarbituric acid‐reactive substances

The TBARS of oil samples was determined on the basis of John et al. (2005). The malondialdehyde (MDA) concentration could be expressed in thiobarbituric acid (TBA) number as below: TBARS (ppm) = sample A_532_ × 2.77.

### 2.6 Prediction of shelf life

The shelf lives of oil samples were calculated by the Rancimat method according to Farhoosh (2007). In this research, 3 g of oil sample, 20 L/h of airflow rate, and four temperatures (80, 90, 100 and 110℃) are selected to measure the RITs of oil samples according to Metrohm's recommendation. Through plotting the linear regression of log RIT versus *t*(Log RIT = *a*(*t*) + *b*), the shelf lives of oil samples at 25℃ could be calculated. The slope of the line yielded temperature coefficients (*T*_coeff_, ℃^−1^) and the ratio of RIT at *t* ℃ and *t* + 10℃ yielded temperature acceleration factor (Q_10_ number).

### 2.7 Statistical analysis

All the experiments were conducted three times. The acquired data were presented as mean ± standard deviation (*SD*). Statistical analysis was performed by SPSS 19.0 software (SPSS Inc.). Differences between means were evaluated by Student–Newman–Keuls (S–N–K) test. *p* values < .05 indicate statistical significance.

## 3 RESULTS

### 3.1 Selection of the best single antioxidant for DHA algae oil and walnut oil

**FIGURE 1:**
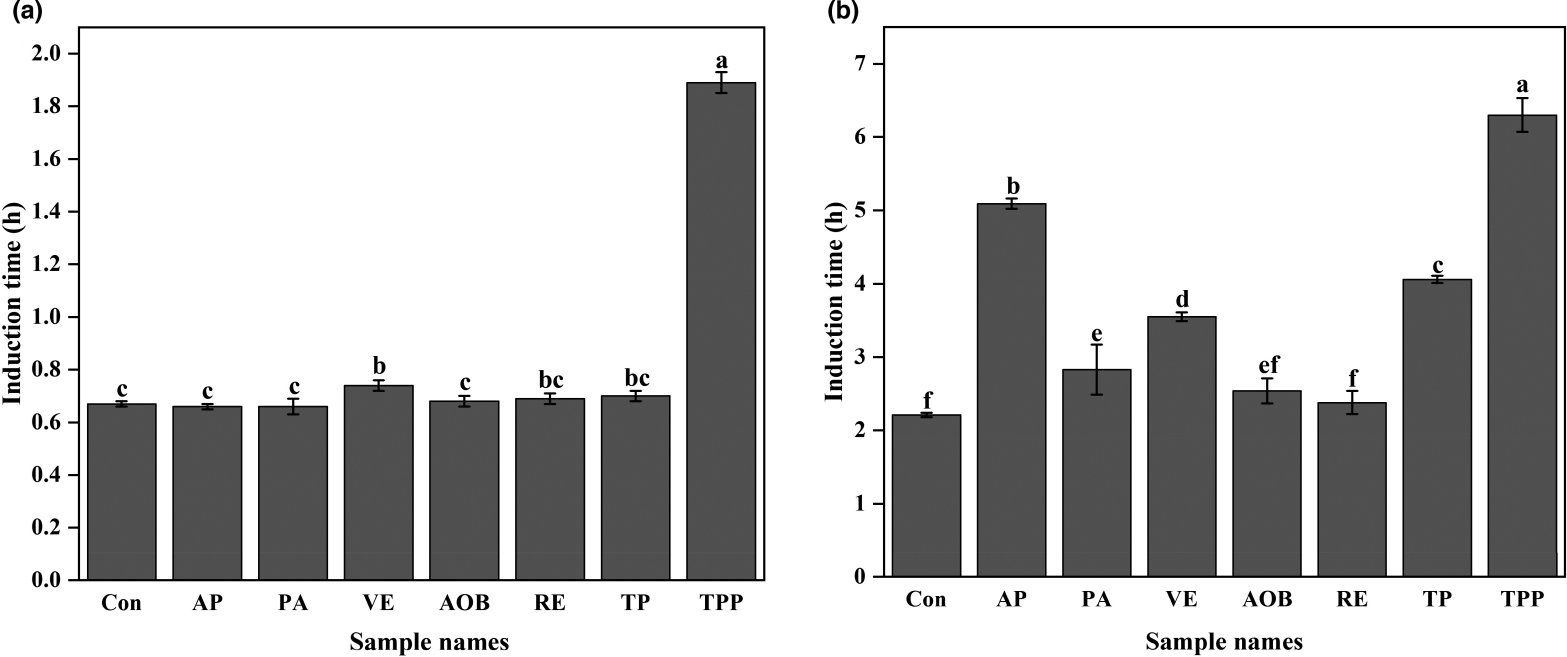
Rancimat induction times (RITs; at 110℃) of docosahexanoic acid (DHA) algae oil (a) and walnut oil (b) containing various single antioxidants including ascorbyl palmitate (AP), phytic acid (PA), vitamin E (VE), antioxidant of bamboo leaves (AOB), rosemary extract (RE), tea polyphenols (TP), and tea polyphenol palmitate (TPP). All experiments were repeated three times. Different letters (a–f) in each panel at same storage time indicate significant differences from each other (*p* < .05)

Rancimat test was applied for evaluating the oxidative stability of DHA algae oil and walnut oil samples with single antioxidants including AP, PA, VE, AOB, RE, TP, and TPP. For the two kinds of oils, the samples containing TPP both have the longest RIT value, indicating TPP was the best single antioxidant for the two oils (Figure 1). Thus, TPP was used to combine with AP, PA, VE, AOB, RE, or TP, respectively, to form antioxidant mixtures to further strengthen the oxidative stability of the two oils.

### 3.2 Optimization of formulations of the antioxidant mixtures for DHA algae oil and walnut oil

**FIGURE 2:**
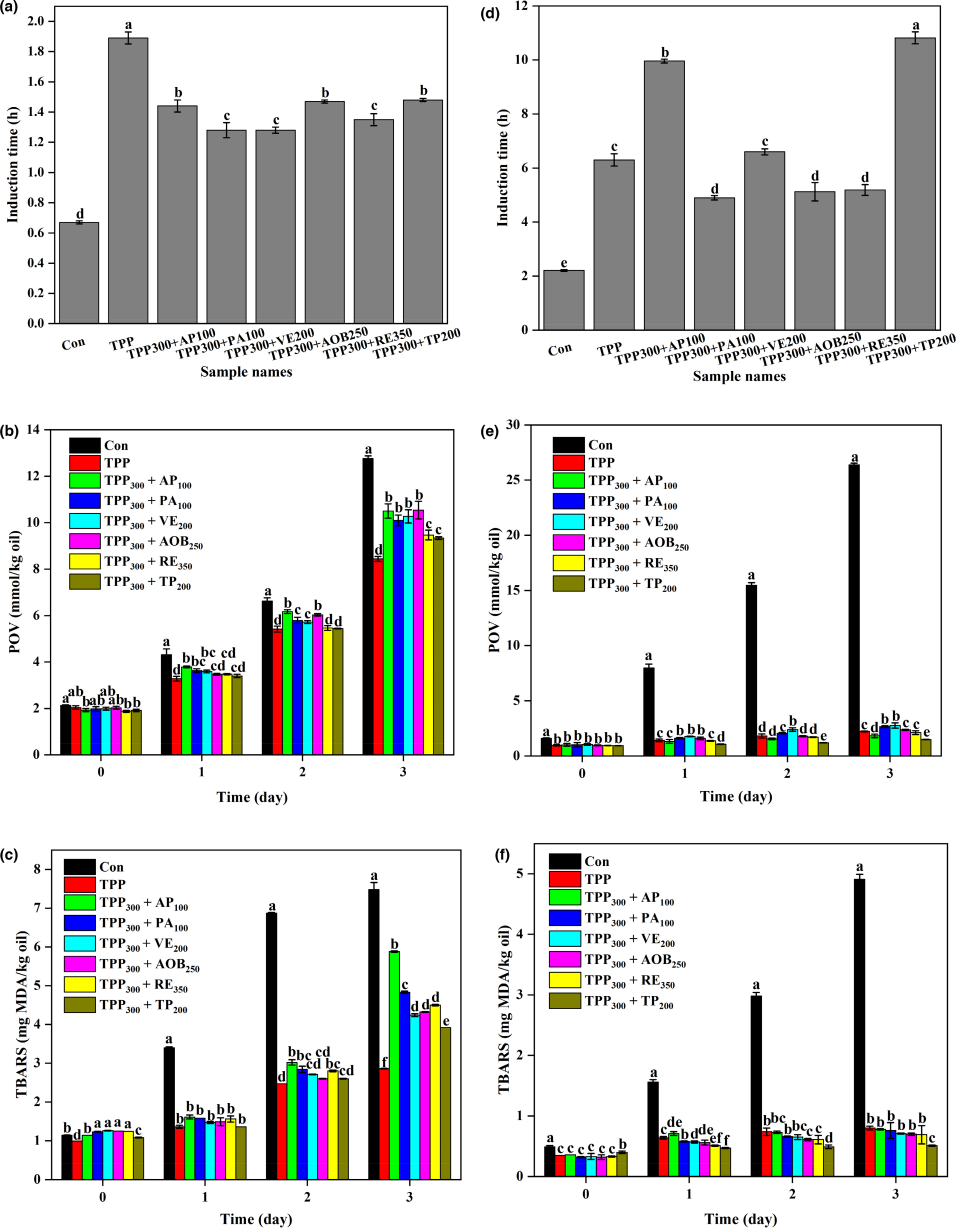
Rancimat induction times (RITs; at 110℃), peroxide values (POVs; at 60℃), and thiobarbituric acid‐reactive substances (TBARS; at 60℃) of docosahexaenoic acid (DHA) algae oil (a–c) and walnut oil (d–f) containing tea polyphenol palmitate (TPP) or the binary mixtures comprised of TPP and one of the other six antioxidants (ascorbyl palmitate (AP), phytic acid (PA), vitamin E (VE), antioxidant of bamboo leaves (AOB), rosemary extract (RE), and tea polyphenols (TP)). All experiments were repeated three times. Different letters (a–f) in each panel at same storage time indicate significant differences from each other (*p* < .05)

For both DHA algae oil and walnut oil, all the binary antioxidant mixtures could significantly prolong the RIT value compared to the control sample (Figure 2). However, the antioxidant effect of all the binary antioxidant mixtures is significantly weaker than the single TPP for DHA algae oil (*p* < .05; Figure 2a), while the combinations of TPP and AP or TP have significantly better antioxidant effects compared to the single TPP in walnut oil (*p* < .05; Figure 2d).

For purpose of verifying the above results further, the changing trends in POV and TBARS values of the oil samples containing TPP and the antioxidant mixtures during accelerated storage were measured. The POV and TBARS values for all the oil samples had progressively risen accompanying the elongation of storage life, symbolizing the generation of primary and secondary oxidation products during accelerated storage (*p* < .05; Figure 2). For DHA algae oil, the samples containing TPP have the lowest levels of POV and TBARS values after every storage period, and the samples containing the combination of TPP and TP have relatively lower levels of POV and TBARS values among all the binary antioxidant mixtures (*p* < .05). For walnut oil, the samples containing the combination of TPP and TP have the lowest levels of POV and TBARS values after every storage period (*p* < .05).

The above‐mentioned results stated that the combination of TPP and TP was the optimal binary mixture for both DHA algae oil and walnut oil. Therefore, the blend ratios of the combination of TPP and TP were optimized for further enhancing the antioxidant efficiency in the two oils.

### 3.3 Optimization of blend ratios of the antioxidant mixtures for DHA algae oil and walnut oil

**FIGURE 3:**
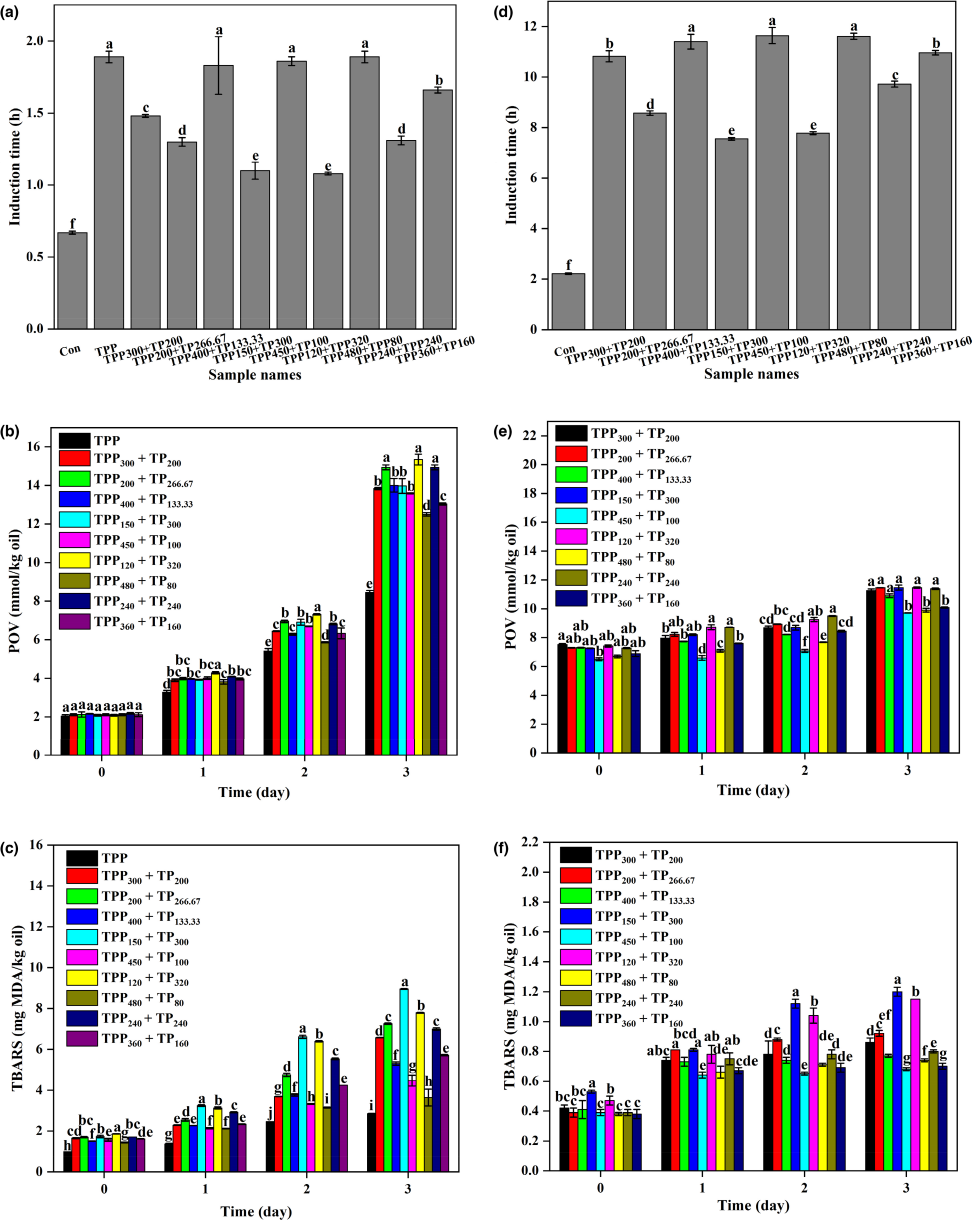
Rancimat induction times (RITs; at 110℃), peroxide values (POVs; at 60℃), and thiobarbituric acid‐reactive substances (TBARS; at 60℃) of DHA algae oil (a–c) and walnut oil (d–f) containing tea polyphenol palmitate (TPP) or the binary mixtures comprised of TPP and tea polyphenols (TP) with different ratios. All experiments were repeated three times. Different letters (a–i) in each panel at same storage time indicate significant differences from each other (*p* < .05)

It could be seen from Figure 3 that the blend ratios of the combination of TPP and TP had a significant effect on the antioxidant effectiveness in the two oils (*p* < .05). For DHA algae oil, the samples containing 480 mg/kg TPP and 80 mg/kg TP have a relatively longer RIT value and lower levels of POV and TBARS values compared to the samples containing other blend ratios (*p* < .05). However, these samples containing the combination of TPP and TP still have significantly shorter RIT value and higher levels of POV and TBARS values than the single TPP (*p* < .05). For walnut oil, the samples containing the combinations of 400 mg/kg TPP and 133.33 mg/kg TP, 450 mg/kg TPP and 100 mg/kg TP, and 480 mg/kg TPP and 80 mg/kg TP have the longest RIT values among all the mixtures with different ratios, while the combination of 450 mg/kg TPP and 100 mg/kg TP is the optimum antioxidant mixtures according to the POV and TBARS assays (*p* < .05).

Therefore, for DHA algae oil, the optimal antioxidant is 600 mg/kg TPP, but for walnut oil, the optimal antioxidant is the combination of 450 mg/kg TPP and 100 mg/kg TP.

### 3.4 Prediction of shelf lives of DHA algae oil and walnut oil

**FIGURE 4:**
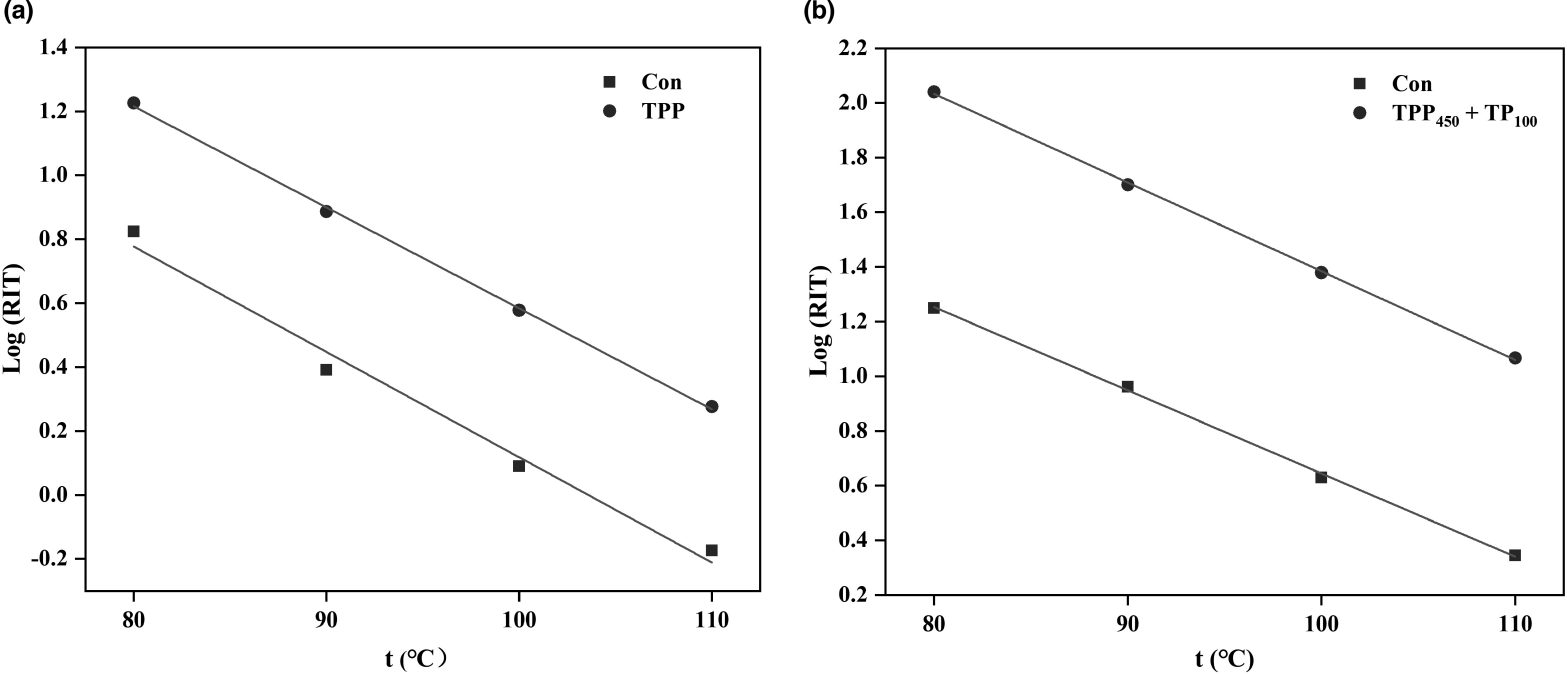
The linear relationship between the natural logarithm of the Rancimat induction time (RIT) and the temperature (t) in docosahexaenoic acid (DHA) algae oil (a) and walnut oil (b). Con was the control DHA algae oil (a) or walnut oil (b) without adding any antioxidants. Tea polyphenol palmitate (TPP) was the DHA algae oil sample containing 600 mg/kg TPP. TPP_450_ + TP_100_ was the walnut oil sample containing 450 mg/kg TPP and 100 mg/kg tea polyphenols (TP)

Rancimat test can also be applied for predicting the shelf life through plotting the logarithms of RITs versus elevated temperatures and extrapolating to room temperature according to Farhoosh (2007). The rate of oxidation increases is accompanied by an increasing temperature. The data calculated from the linear regression of the natural logarithm of the RIT (Log RIT) versus the temperature (*t*) are shown in Figure 4, Tables 1 and 2. The temperature coefficients (*T*_coeff_) of the oils are all within the range from −2.78 × 10^−2^ to −3.50 × 10^−2^℃^−1^ for vegetable oils reported by García‐Moreno et al. (2013) and Q_10_ numbers are all close to 2.00, meaning that an increase of 10℃ approximately halves the RIT of the oil. The results declared that the DHA algae oil sample containing 600 mg/kg TPP had 2.31‐fold longer shelf life than the control DHA algae oil, while the walnut oil sample containing 450 mg/kg TPP and 100 mg/kg TP had 7.74‐fold longer shelf life than the control walnut oil.

**TABLE 1:** Shelf life (Rancimat induction time at 25℃, RIT_25_), temperature coefficients (*T*_coeff_), and Q_10_ numbers (increase of reaction rate due to a 10℃ temperature rise) of docosahexaenoic acid (DHA) algae oil

Sample	Temperatures	RIT (h)	Log (RIT) = *a*(*t*) + *b*			*T*_coeff_ (10^−2^, ℃^−1^)	RIT_25_ (day)	Q_10_
			*a*	*b*	*R* ^2^			
Con	80℃	6.67 ± 0.43	−0.033	3.4132	.9862	−3.30	16.14	2.18 ± 0.47
	90℃	2.46 ± 0.06						
	100℃	1.23 ± 0.03						
	110℃	0.67 ± 0.01						
TPP	80℃	16.84 ± 0.11	−0.0316	3.7424	.9992	−3.16	37.34	2.07 ± 0.10
	90℃	7.70 ± 0.25						
	100℃	3.78 ± 0.10						
	110℃	1.89 ± 0.04						

Note

Con was the control DHA algae oil without adding any antioxidants. TPP was the DHA algae oil with 600 mg/kg tea polyphenol palmitate.

**TABLE 2:** Shelf life (Rancimat induction time at 25℃, RIT_25_), temperature coefficients (*T*_coeff_), and Q_10_ numbers (increase of reaction rate due to a 10℃ temperature rise) of walnut oil

Sample	Temperatures	RIT (h)	Log (RIT) = *a*(*t*) + *b*			*T*_coeff_ (10^−2^, ℃^−1^)	RIT_25_ (day)	Q_10_
			*a*	*b*	*R* ^2^			
Con	80℃	17.74 ± 1.07	−0.0304	3.688	.9991	−3.04	35.30	2.00 ± 0.12
	90℃	9.16 ± 0.08						
	100℃	4.27 ± 0.12						
	110℃	2.21 ± 0.03						
TPP_450_ + TP_100_	80℃	109.78 ± 8.24	−0.0325	4.6291	.9997	−3.25	273.14	2.11 ± 0.07
	90℃	50.15 ± 2.45						
	100℃	23.91 ± 0.14						
	110℃	11.64 ± 0.32						

Note

Con was the control walnut oil without adding any antioxidants. TPP_450_ + TP_100_ was the walnut oil with 450 mg/kg tea polyphenol palmitate and 100 mg/kg tea polyphenols.

## 4 DISCUSSION

Edible oils high in PUFAs are very susceptible to lipid oxidation, which can cause off‐flavors and increase the incidence of hepatic inflammation and then lead to health issues (Großhagauer et al., 2019). In our study, seven kinds of antioxidants involving AP, PA, VE, AOB, RE, TP, and TPP (Authorized by Chinese Standard GB 2760‐2014 in edible oils) were applied in both DHA algae oil and walnut oil. Among them, AP can scavenge activated oxygen species (Satyanarayana et al., 2000), PA can suppress iron‐catalyzed oxidative reactions (Graf & Eaton, 1990), and phenolic compounds including VE, AOB, RE, TP, and TPP can block free radical chain reaction (Hunyadi, 2019; Yan et al., 2020).

Our results indicated that TPP revealed the strongest efficiency among all single antioxidants both in DHA algae oil and walnut oil. Bulk oils are mainly composed of triacylglycerols, which are often seen as a simple homogeneous medium. Therefore, TPP, as a lipophilic TP fatty acid ester, can be fully soluble in oil, which is beneficial to exert antioxidant functions (Luo et al., 2017; Olajide et al., 2020). Our results also found that the combination of TPP and TP exerted the best effectiveness among all the binary antioxidant mixtures. This is probably because, in fact, there are also many minor components like phospholipids, free fatty acids, and polar products generated from lipid oxidation in oils (Chen et al., 2011). Much evidence shows that these components can combine with trace water in oils to form physical structures such as association colloids which are possibly the site of lipid oxidation (Laguerre et al., 2015; Villeneuve et al., 2021). TP is more hydrophilic and has a better affinity for the interface of association colloids compared to TPP (Laguerre et al., 2015).

The above‐mentioned results presented that the optimal antioxidant for DHA algae oil (the single TPP) is different from that for walnut oil (the combination of TPP and TP). This phenomenon is closely related to the pretreatment and refining processes of raw oils (Chen et al., 2011). Before refining, crude DHA algae oil needs to be fermented from microalgae and then obtained after cell lysis and centrifugation (Yin et al., 2018), while crude walnut oil can be obtained from walnut kernels (60%–65% levels of oil) directly (González‐Gómez et al., 2019). It can be seen that the extraction process of DHA algal oil is complex and tedious than that of walnut oil, which may result in its higher concentration of such minor components, and then requires a stricter refining process further. Meanwhile, the difference of fatty acid compositions between DHA algae oil and walnut oil also leads to difference in refining process (Vaisali et al., 2015). In detail, DHA algae oil has a higher content of PUFAs resulting in more levels of free fatty acids than walnut oil, thus, the step of deacidification of DHA algae oil is stricter than that of walnut oil (Vaisali et al., 2015). As a result, DHA algae oil may have a relatively lower amount of minor components such as phospholipids, free fatty acids, and polar products than walnut oil due to its stricter refining process. Therefore, the combination of TP with TPP cannot exert synergistic effects in DHA algae oil because TP cannot play its good role on the surface of association colloids comprised of minor components, while the opposite in walnut oil.

The Rancimat test was first established by Hadorn and Zurcher (1974) and then applied to evaluate the oxidative stability of oils as an accelerated analysis. Reynhout (1991) proposed that since the rate of lipid oxidation is closely related to temperature, the RIT value of oils decreases with increasing temperature logarithmically. Therefore, Farhoosh et al. (2008) indicated that the linear regression model between Log RIT versus temperature can be selected to estimate the shelf life of oils as RIT_25_. In detail, the RIT values are measured under four temperatures, and then the fitting line of Log RIT versus temperature is plotted as Log RIT = *a*(*t*) + b. At the end, the shelf life of oils is calculated by substituting *t* = 25℃ into the equation. Although there are some uncertainties due to the decrease of the solubility of oxygen by almost 25% with each 10℃ increase of temperature, it can still be found that adding the optimal antioxidant to DHA algae oil and walnut oil, respectively, can significantly lengthen the shelf lives of the two oils (Liu et al., 2018; Reynhout, 1991). However, our results showed that the optimal antioxidants for DHA algae oil and walnut oil, respectively, showed different effects on prolonging the shelf lives of them. This is because the oxidative susceptibility of DHA algae oil is more sensitive than that of walnut oil as well as its higher initial oxidation degree than that of walnut oil (Figures 1 and 2), which causes more difficulty for the antioxidants to act on DHA algae oil against lipid oxidation.

## 5 CONCLUSION

The results of the Rancimat, POV, and TBARS assays all indicated that TPP was the most effective single antioxidant both in DHA algae oil and walnut oil. Through optimizing formulations and blend ratios of the antioxidant mixtures, it could be found that the optimal antioxidant for DHA algae oil is 600 mg/kg TPP and the optimal composite antioxidant for walnut oil is the combination of 450 mg/kg TPP and 100 mg/kg TP. The optimal antioxidant could prolong the shelf life of DHA algae oil and walnut oil by 2.31‐ and 7.74‐fold, respectively.

## CONFLICT OF INTEREST

The authors declare that they have no conflict of interest.

## Supporting information

Table S1

## Data Availability

Research data are not shared.
