# Supplementary material for: Effects of different antioxidants and their combinations on the oxidative stability of DHA algae oil and walnut oil
Source: Food Sci Nutr. 2022 Apr 11;10(8):2804–12. doi: 10.1002/fsn3.2883 (PMC9361454; doi:10.1002/fsn3.2883)
Supplement: Supplementary file 1 — Table S1 [file FSN3-10-2804-s001.docx]

Table 1S. Types and amounts of single antioxidants and their binary mixtures added to DHA algae oil and walnut oil

| Sample name* | Antioxidant type | Total amounts added (mg/kg) |
| --- | --- | --- |
| Con | Without antioxidant | 0 |
| AP | Ascorbyl palmitate | 200 |
| PA | Phytic acid | 200 |
| VE | Vitamin E | 400 |
| AOB | Antioxidant of bamboo leaves | 500 |
| RE | Rosemary extract | 700 |
| TP | Tea polyphenols | 400 |
| TPP | Tea polyphenol palmitate | 600 |
| TPP_300_ + AP_100_ | Tea polyphenol palmitate + ascorbyl palmitate | 400 |
| TPP_300_ + PA_100_ | Tea polyphenol palmitate + phytic acid | 400 |
| TPP_300_ + VE_200_ | Tea polyphenol palmitate + vitamin E | 500 |
| TPP_300_ + AOB_250_ | Tea polyphenol palmitate + antioxidant of bamboo leaves | 550 |
| TPP_300_ + RE_350_ | Tea polyphenol palmitate + rosemary extract | 650 |
| TPP_300_ + TP_200_ | Tea polyphenol palmitate + tea polyphenols | 500 |
| TPP_200_ + TP_266.67_ | Tea polyphenol palmitate + tea polyphenols | 466.67 |
| TPP_400_ + TP_133.33_ | Tea polyphenol palmitate + tea polyphenols | 533.33 |
| TPP_150_ + TP_300_ | Tea polyphenol palmitate + tea polyphenols | 450 |
| TPP_450_ + TP_100_ | Tea polyphenol palmitate + tea polyphenols | 550 |
| TPP_120_ + TP_320_ | Tea polyphenol palmitate + tea polyphenols | 440 |
| TPP_480_ + TP_80_ | Tea polyphenol palmitate + tea polyphenols | 560 |
| TPP_240_ + TP_240_ | Tea polyphenol palmitate + tea polyphenols | 480 |
| TPP_360_ + TP_160_ | Tea polyphenol palmitate + tea polyphenols | 520 |

* The subscripts refer to the level of antioxidant used in mg/kg of oil.
